# Exercise and cancer mortality in Korean men and women: a prospective cohort study

**DOI:** 10.1186/s12889-018-5669-1

**Published:** 2018-06-19

**Authors:** Yongho Jee, Youngwon Kim, Sun Ha Jee, Mikyung Ryu

**Affiliations:** 1 Department of Public Health, Seoul National University, Seoul, Korea; 2 Department of Health, Kinesiology, and Recreation, College of Health, University of Utah, Salt Lake City, UT USA; 3 MRC Epidemiology Unit, University of Cambridge School of Clinical Medicine, Cambridge, UK; 4 Institute for Health Promotion, Graduate School of Public Health, Yonsei University, Seoul, Korea; 5 Institute on Aging, Ajou University Medical Center, Suwon, Korea; 6 Central College, Kyonggi University, Suwon, Korea

**Keywords:** Exercise, cancer, death, cohort

## Abstract

**Background:**

Little is known about longitudinal associations of exercise with different types of cancer, particularly in Asian populations. The purpose of this research was to estimate the association between the duration of exercise and all-cause and cancer-specific mortality.

**Method:**

Data were obtained from the Korean Metabolic Syndrome Mortality Study (KMSMS), a prospective cohort study of 303,428 Korean adults aged 20 years or older at baseline between 1994 and 2004 after exclusion of individuals with missing variables on smoking and exercise. Death certificate-linked data until 31 December 2015 were provided by the Korean National Statistical Office. Cox regression models were constructed to evaluate the associations of exercise with cancer mortality after adjusting for potential confounders such as age, alcohol consumption and smoking status.

**Results:**

During the follow-up period of 15.3 years (4,638,863 person-years), a total of 16,884 participants died. Both men and women who exercised showed approximately 30% decreased hazards of mortality, compared to those who did no exercise (hazard ratio (HR) 0.70, 95% confidence interval (CI)=0.68-0.73 for men, HR=0.71, CI : 0.67-0.75). A notable observation of this study is the curvilinear associations between the total duration of exercise per week and cancer mortality, with the lowest risk being observed at the low-to-medium levels of exercise; this trend of associations was found for esophagus, liver, lung, and colorectal cancer mortality in men, and all-cause, all-cancer and lung cancer mortality in women.

**Conclusions:**

Individuals who exercised showed considerably lower all-cause and cancer mortality risks compared with those who did no exercise. Policies and clinical trials aimed at promoting minimal or moderate participation in exercise may minimize cancer mortality risk.

**Electronic supplementary material:**

The online version of this article (10.1186/s12889-018-5669-1) contains supplementary material, which is available to authorized users.

## Background

Cancer is one of the leading causes of death worldwide. Globally, cancer accounts for approximately 8.2 million deaths in 2012, with more than 14 million new cancers diagnosed. [1]. In Korea, 180,530 deaths are predicted for all cancers excluding non-melanoma skin cancer in 2035, with more than 375,250 new cancers diagnosed [1].

According to expert review by Anand et al, up to almost 95% of cancer events are attributable to lifestyle factors such as physical inactivity, cigarette smoking, poor diet, alcohol, and obesity, and thus are potentially preventable [2]. Physical activity is defined as any bodily movement produced by skeletal muscles that results in energy expenditure, while exercise is a subcategory of physical activity, which is defined as a planned, repetitive, and purposive in the sense that improvement or maintenance of one or more components of physical fitness is an objective [3].

Association between exercise duration and cancer mortality was introduced by previous studies. According to large meta-analysis conducted by Li et al, high level of physical activity lowered the risk of cancer mortality in the general population and cancer survivors compared to inactivity, specifically individuals underwent the highest levels of physical activity had a 17% reduction in cancer mortality [4].

Convincing evidence indicates that regular participation in exercise is associated with reduced risk of mortality [5, 6], a recent meta-analysis study found regular physical activity to be protective against a wide variety of types of cancer incidence, using data pooled from 12 large prospective cohort studies comprising over 1.4 million people (with nearly 190,000 cases of cancer) from the United States and Europe [7].

A few other cohort studies [8–12] have also demonstrated inverse associations between physical activity and cancer risk, but all prior research have used data of individuals from Western countries.

Moon et al have reported that Asians have a lower body mass index (BMI) but higher percentage body fat than whites, and have a different fat distribution from whites [13]. Wang et al. reported that Asians had more upper body subcutaneous fat than did whites [14]. Therefore, increased health risks associated with obesity appear to occur at a lower BMI in Asians. In the context of physical activity, relatively low levels of leisure time physical activity and exercise among South Asian people were reported [15]. Nonetheless, a few previous studies investigating physical activity in relation to cancer in Asian populations were either cross-sectional or had low statistical power due to the small sample size [16–19]. Therefore, the purpose of this research was to examine the associations of exercise per week with all-cause and cancer-specific mortality using data from a large-scale prospective cohort study of Koreans.

## Methods

### Study population

Data were obtained from the Korean Metabolic Syndrome Mortality Study (KMSMS), a prospective cohort study of 560,543 Korean adults aged 20 years or older at baseline between 1994 and 2004 [19]. After the exclusion of individuals with missing variables on smoking and exercise, a final sample of 303,428 participants were included in the present analysis (171,594 men and 131,834 women).

### Data collection

Demographic information, smoking status (never, past, or current), and a medical history of comorbidities including atherosclerotic cardiovascular disease and cancer were assessed by written questionnaires. Information on exercise was obtained by asking “Do you exercise regularly?” (Yes/No) If participants answered “Yes,” they were asked to indicate the frequency of exercise per week in on a continuous scale, which we then categorized into 4 comparison groups (0, 1-2 times, 3-4 times, or ≥5 times per week). The next question asked about average hours and minutes spent per exercise session. We multiplied the exercise frequency per week by the average time spent per exercise session (hours and minutes) to obtain the total duration (i.e. overall volume) of exercise per week (<100, 100-249, 250-399, ≥400 minutes/week). We suggested the type of exercise to the participants; jogging, freehand exercise, walk, hiking, fitness, yoga, swimming, aerobics, golf, jump rope, etc.

Weight and height measurements were taken while participants were wearing light clothing. Body mass index (BMI) was calculated as weight in kilograms divided by the square of height in meters.

### Mortality follow-up

Death certificate-linked data until 31 December 2015 were provided by the Korean National Statistical Office. Mortality outcomes were ascertained from the causes of death listed on death certificates. A computerized search of death certificate data from the National Statistical Office in Korea was performed, using unique identification numbers assigned at birth. Cancer mortality was defined as any deaths due to cancer using the primary cause of death based on the International Classification of Diseases Tenth Revision codes C00-C97; codes used to classify specific types of cancer mortality are provided in the Additional file 1: Table S1.

### Statistical analysis

Descriptive analyses were performed to summarize the means and standard deviations for continuous variables and percentages for categorical variables in men and women separately. Age-standardized cause-specific mortality rates were calculated across these categories using the Korean Population and Housing Census data in 2010 as a standard population.

Cox regression models were used to estimate the association between exercise duration and cancer with adjustment for potential confounders (age, alcohol intake [yes/no], and smoking status [non-smoker, ex-smoker, current smoker]). Proportional assumption was tested by using Schoenfeld residuals. The survival curve according to exercise level was plotted using the life-table method. To calculate attributable fractions of non-exercise for mortality, individuals who reported that they exercised served as the reference group. All analyses were performed using SAS version 9.4. All statistical tests were two-sided, and statistical significance was determined at an alpha level of 5%.

## Results

The average (standard deviation) age of KMSMS subjects was 46.0 (10.6) years in men and 46.2 (11.3) years in women. Table 1 shows characteristics of the study participants by exercise categories. Compared to those who did not exercise, those who exercised were more likely to have higher age, BMI, and drinking rates or lower smoking rates.

**Table 1:** Baseline Characteristics of the Korean Metabolic Syndrome Mortality Study

	No exercise	Exercise	Total duration of exercise per week (minutes/week)^a^		
			<150	150-299	≥300
	(N=162,863)	(N=140,565)	(N=47,579)	(33,729)	(29,102)
Age, y, mean (SD)	44.7 (11.2)	47.7 (10.3)	46.1 (10.0)	47.6 (10.2)	49.7 (10.3)
Body mass index, kg/m^2^, mean (SD)	23.5 (3.2)	24.0 (2.8)	23.7 (2.9)	23.8 (2.8)	23.9 (2.7)
Alcohol drinking, g /day	12.3 (27.2)	13.8 (26.8)	14.0 (26.2)	13.1 (26.2)	14.0 (28.4)
Average frequency per week	0	3.2 (2.0)	2.1 (1.5)	3.6 (1.6)	4.8 (1.8)
Average minutes per exercise (minutes)	0	74.4 (65.0)	48.0 (40.8)	76.2 (60.7)	119.7 (81.6)
Total duration of exercise (minutes/week)	0	218.3 (202.0)^a^	74.8 (37.8)	203.0 (35.6)	469.0 (219.9)
Condition, %					
Sex (male)	51.6	62.3	66.4	59.2	59.3
Alcohol drinking (yes)	59.2	68.4	71.4	66.2	65.6
Smoking status (former)	12.7	23.1	22.0	23.3	25.7
Smoking status (current)	34.0	29.3	32.7	26.2	25.7

^a^30,155 with missing

During the follow-up period of 15.3 years (4,638,863 person years), a total of 16,884 (11,919 men and 4,965 among women) participants died, providing sufficient statistical power to examine the association between exercise and cancer mortality. Table 2 shows age adjusted mortality rates and hazard ratios (HR) of all-cause, cancers, and cancer types by exercise status in men. Men who exercised had lower all-cause and all-cancer mortliaty rates per 100,000 person-years compared with those who did not exericse. Compared with men who did no exercise, those who exercised showed 0.70 (95% confidence interval [CI]: 0.68-0.73) and 0.77 (95% CI: 0.73-0.81) times lower hazards of all-cause and all-cancer mortality, respectively. In addition, men who exercised showed lower hazards of esophageal (HR=0.67; 95% CI: 0.47-0.97, liver (HR=0.83 95% CI: 0.74-0.94), lung (HR=0.71 95% CI: 0.64-0.79), colorectal (HR=0.65 95% CI: 0.53-0.80), and stomach cancer (HR=0.62 95% CI: 0.53-0.73) than those who did not exercise. When men who exercised were set as the reference, those who did no exercise showed 1.42 (95% CI: 1.37-1.48) times higher risk of mortality. Non-exercise prevalence was found to be 53.7%, while attributable fractions of non-exercise for all-cause and all-cancer mortality were 18.4% and 13.9%, respectively.

**Table 2:** Age-adjusted Mortality Rates per 100,000 and Hazard Ratios (HR) for Death Due to All Causes, All Cancers, and Various Cancers by total duration (minutes/week) of Exercise per Week at Baseline in Korean men, 1994-2015

	No exercise	Exercise	Total duration of exercise per week (minutes/week)			P for linear trend
			<150	150-299	≥300	
All causes						
Number of death	6,150	5,769	1,668	1,215	1,355	
Death rate	572.7	389.3	371.7	376.8	384.2	
HR (95% CI)	1.0	0.70 (0.68 – 0.73)	0.69 (0.65-0.73)	0.67 (0.63-0.71)	0.67 (0.63-0.71)	<.0001
All cancers						
Number of death	2,739	2,740	781	611	640	
Death rate	233.2	166.4	150.5	165.5	163.3	
HR (95% CI)	1.0	0.77 (0.73 – 0.81)	0.74 (0.68-0.80)	0.78 (0.71-0.85)	0.73 (0.67-0.80)	<.0001
Esophagus						
Number of death	65	56	16	15	13	
Death rate	5.6	3.2	4.0	4.1	2.7	
HR (95% CI)	1.0	0.67 (0.47 – 0.97)	0.64 (0.37-1.12)	0.84 (0.48-1.48)	0.66 (0.36-1.21)	0.1426
Head and Neck						
Number of death	37	32	14	3	7	
Death rate	3.0	1.7	2.2	0.7	1.3	
HR (95% CI)	1.0	0.67 (0.41 – 1.09)	0.98 (0.53-1.83)	0.28 (0.09-0.93)	0.60 (0.26-1.36)	0.0595
Liver						
Number of death	551	573	163	130	132	
Death rate	40.5	31.0	26.4	34.3	29.1	
HR (95% CI)	1.0	0.83 (0.74 – 0.94)	0.76 (0.64-0.90)	0.85 (0.70-1.03)	0.83 (0.68-1.00)	0.0183
Lung						
Number of death	745	650	173	128	171	
Death rate	70.7	39.2	33.2	36.0	42.6	
HR (95% CI)	1.0	0.71 (0.64 – 0.79)	0.65 (0.55-0.77)	0.64 (0.53-0.77)	0.75 (0.63-0.89)	<.0001
Colorectal						
Number of death	203	185	60	42	39	
Death rate	17.0	11.6	10.3	12.1	9.7	
HR (95% CI)	1.0	0.65 (0.53 – 0.80)	0.72 (0.54-0.96)	0.66 (0.47-0.92)	0.55 (0.39-0.78)	0.0001
Pancreas						
Number of death	172	252	73	69	50	
Death rate	13.7	14.5	14.4	17.1	11.5	
HR (95% CI)	1.0	1.0 (1.00 – 1.00)	1.11 (0.84-1.46)	1.44 (1.08-1.90)	0.96 (0.69-1.32)	0.3910
Kidney						
Number of death	31	42	12	9	11	
Death rate	2.3	3.3	2.0	3.1	7.0	
HR (95% CI)	1.0	0.95 (0.59 – 1.53)	0.93 (0.47-1.81)	0.92 (0.44-1.95)	1.01 (0.50-2.04)	0.9846
Stomach						
Number of death	347	287	89	63	64	
Death rate	31.8	17.9	18.6	15.3	16.1	
HR (95% CI)	1.0	0.62 (0.53 – 0.73)	0.65 (0.52-0.83)	0.62 (0.47-0.81)	0.56 (0.43-0.74)	<.0001
Prostate						
Number of death	64	84	22	20	21	
Death rate	7.0	6.6	5.8	6.7	6.1	
HR (95% CI)	1.0	0.80 (0.57 – 1.11)	0.80 (0.49-1.31)	0.85 (0.51-1.41)	0.72 (0.44-1.19)	0.1641

Adjusted for age, alcohol drinking status (non, and current), and smoking status (never, former, and current)

In addition, Table 2 shows associations between the total duration of exercise per week and mortality from all causes, cancer, and specific types of cancer in men. Regarding all-cause mortality, the exercise group showed lower rates (HR=0.69 for <150 minutes per week; HR=0.67 for 150-299 minutes per week; HR=0.67 for ≥300 minutes per week) than the non-exercise group. Analyses for all-cancer mortality showed similar rates of HR=0.74 (<150 minutes per week), HR=0.78 (150-299 minutes per week), and HR=0.73 (≥300 minutes per week).

Table 3 presents age-adjusted mortality rates and hazard ratios (HR) of all-cause, cancers, and cancer types by exercise status in women. Likewise, women who exercised had lower all-cause and all-cancer mortliaty rates per 100,000 person-years compared with those who did not exericse. Table 3 also shows associations between the total duration of exercise per week and mortality from all causes, cancer, and specific types of cancer in women. Regarding all-cause mortality, the exercise group showed lower rates (HR=0.73 for <150 minutes per week; HR=0.65 for 150-299 minutes per week; HR=0.69 for ≥300 minutes per week) than the non-exercise group.

**Table 3:** Age-adjusted Mortality Rates per 100,000 and Hazard Ratios (HR) for Death Due to All Causes, All Cancers, and Various Cancers by total duration (minutes/week) of Exercise per Week at Baseline in Korean women, 1994-2015

	No exercise	Exercise	Total duration of exercise per week (minutes/week)			P for linear trend
			<150	150-299	≥300	
All causes						
Number of death	3,345	1,619	472	367	356	
Death rate	267.9	242.3	240.4	220.9	231.2	
HR (95% CI)	1.0	0.71 (0.67 – 0.75)	0.73 (0.66-0.80)	0.65 (0.58-0.72)	0.69 (0.61-0.76)	<.0001
All cancers						
Number of death	1,320	740	201	174	156	
Death rate	86.2	92.6		93.9	88.2	84.5
HR (95% CI)	1.0	0.81 (0.74 – 0.89)	0.78 (0.67-0.90)	0.76 (0.65-0.89)	0.75 (0.63-0.88)	<.0001
Esophagus						
Number of death	3	2	0	1	0	
Death rate	0.6	0.2				
HR (95% CI)	1.0	0.83 (0.14 – 5.07)	NE	NE	NE	NE
Head and Neck						
Number of death	9	8	2	2	0	
Death rate	0.9	1.7				
HR (95% CI)	1.0	1.26 (0.48 – 3.28)	NE	NE	NE	NE
Liver						
Number of death	152	95	28	18	25	
Death rate	8.5	12.7	17.2	6.1	14.4	
HR (95% CI)	1.0	0.90 (0.70 – 1.17)	0.94 (0.63-1.41)	0.69 (0.42-1.12)	1.04 (0.68-1.59)	0.5412
Lung						
Number of death	242	120	35	28	20	
Death rate	16.4	15.2	15.2	13.8	10.2	
HR (95% CI)	1.0	0.74 (0.59 – 0.92)	0.76 (0.53-1.08)	0.69 (0.47-1.02)	0.54 (0.34-0.85)	0.0013
Colorectal						
Number of death	137	79	19	22	13	
Death rate	9.3	8.9	8.5	8.4	7.4	
HR (95% CI)	1.0	0.83 (0.63 – 1.10)	0.71 (0.44-1.14)	0.92 (0.59-1.45)	0.60 (0.34-1.05)	0.0801
Pancreas						
Number of death	119	75	22	15	20	
Death rate	6.8	7.4	7.6	5.5	9.6	
HR (95% CI)	1.0	0.91 (0.68 – 1.21)	0.94 (0.60-1.49)	0.73 (0.47-0.81)	1.06 (0.66-1.71)	0.7405
Kidney						
Number of death	7	3	0	0	1	
Death rate	0.2	0.3	0.0	0.0	0.5	
HR (95% CI)	1.0	0.51 (0.13 – 1.98)	NE	NE	NE	NE
Stomach						
Number of death	143	62	13	17	12	
Death rate	11.7	8.2	7.3	9.1	6.8	
HR (95% CI)	1.0	0.62 (0.46 – 0.83)	1.30 (0.80-2.12)	0.82 (0.44-1.55)	0.93 (0.50-1.76)	0.0036
Breast						
Number of death	79	56	20	11	11	
Death rate	6.1	6.4	8.3	4.2	6.6	
HR (95% CI)	1.0	1.06 (0.75 – 1.50)	0.65 (0.52-0.83)	0.62 (0.47-0.81)	0.56 (0.43-0.74)	0.7654
Cervix						
Number of death	26	14	4	2	2	
Death rate	1.3	2.6	1.6	2.1	0.7	
HR (95% CI)	1.0	0.74 (0.39 – 1.42)	0.74 (0.26-2.13)	0.42 (0.10-1.78)	0.46 (0.11-1.95)	0.1234

Adjusted for age, alcohol drinking status (non, and current), and smoking status (never, former, and current)

**Fig. 1:**
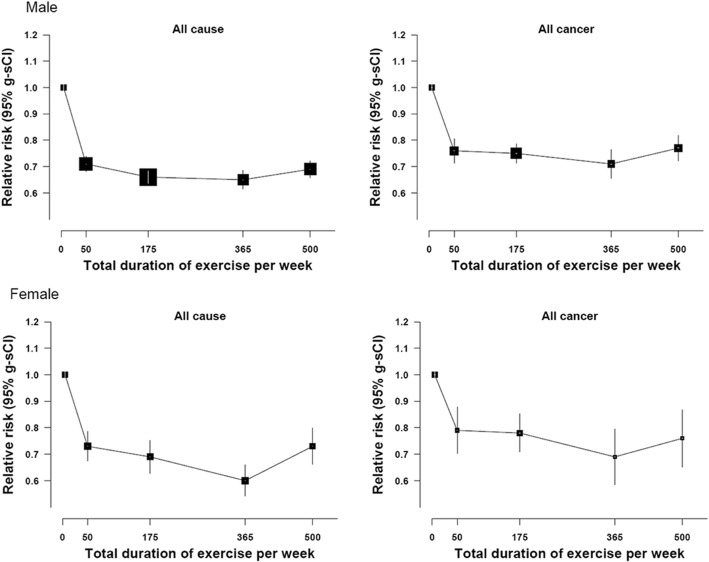
Hazard ratios and 95% group specific confidence intervals of all causes and all cancer mortality, the Korean Metabolic Syndrome Mortality Study

Table 4 shows associations between the total duration of exercise per week with different categories and mortality from all causes and all cancers in men and women. We found that those who did at least 100 minutes per week showed significant reduction and reduced until less than 400 minutes per week in men and women. In other words, the largest reduction in all cause and all cancer mortality risk was observed in the minimal or middle levels of duration of exercise per week rather than in the highest categories, providing J-shaped associations. In general, the intermediate levels of exercise duration (i.e. categories of 100-249min and 250-399min) showed the lowest (statistically significant) HR values for all-cause, all-cancer, and lung; the HRs in the highest duration category were slightly higher (although not significantly for all causes and all cancer (Fig. 1).

**Table 4:** Age-adjusted Mortality Rates per 100,000 and Hazard Ratios (HR) for Death Due to All Causes, All Cancers, and Various Cancers by total duration (minutes/week) of Exercise per Week at Baseline in Korean men and women, 1994-2015

	No exercise	Total duration of exercise per week (minutes/week)				P for linear trend
		<100	100 - 249	250 - 399	≥400	
Men						
All causes						
Number of death	6,150	1,162	1,562	644	870	
Death rate	572.7	383.4	370.5	370.7	391.8	
HR (95% CI)	1.0	0.71 (0.67 – 0.76)	0.66 (0.63 – 0.70)	0.65 (0.60 – 0.71)	0.69 (0.64 – 0.74)	<.0001
All cancers						
Number of death	2,739	547	767	302	416	
Death rate	233.2	158.4	159.4	142.6	179.9	
HR (95% CI)	1.0	0.76 (0.70 – 0.84)	0.75 (0.70 – 0.81)	0.71 (0.63 – 0.80)	0.77 (0.70 – 0.85)	<.0001
Women						
All causes						
Number of death	3,346	317	498	173	212	
Death rate	267.9	220.5	242.9	207.5	235.9	
HR (95% CI)	1.0	0.73 (0.65 – 0.82)	0.69 (0.63 – 0.76)	0.60 (0.51 – 0.69)	0.73 (0.63 – 0.84)	<.0001
All cancers						
Number of death	1,320	139	223	82	87	
Death rate	86.2	94.1	90.3	82.9	86.4	
HR (95% CI)	1.0	0.79 (0.67 – 0.94)	0.78 (0.68 – 0.90)	0.69 (0.55 – 0.87)	0.76 (0.61 – 0.94)	0.0046

## Discussion

To the best of our knowledge, this study is the first investigation examining the longitudinal associations of exercise with various cancer mortality outcomes in Asian adult populations. We found that over 15.3-year follow-up (4,638,863 person-years; 16,884 deaths) both men and women who exercised showed over 20% decreased hazards of all-cause and all-cancer mortality, respectively, compared to those who did no exercise. A notable observation of this study is the curvilinear associations between the total duration of exercise and cancer including all-cause, all-cancer, esophagus, lung, colorectal, and stomach cancer in men and all cause, all cancer and lung cancer in women. This finding specifically indicates that risk of cancer mortality was not lowest in the highest level of exercise, but in the minimal or intermediate level of exercise, suggesting that public and clinical efforts may not necessarily need to focus on encouraging people to participate in maximal levels of exercise in order to minimize cancer risk. Since our study merely analyzed the association in exercise, further study is required in order to examine whether the similar J-shape appears in the moderate type of physical activity like active walking, commuting activity, and household chores.

A recent systematic review showed the continuous does-response relations between physical activity and cancer including 35 articles for breast cancer and 19 for colon cancer from the study on Global Ageing and Adult health conducted in China, Ghana, India, Mexico, Russia, and South Africa and US National Health and Nutrition Examination Surveys [20]. People who achieve total physical activity levels several times higher than 150 minutes of moderate-to-vigorous physical activity per week as recommended by World Health Organization [6, 21] showed a significant reduction in the risk of cancers studied [17]. Our results showed that individuals who accumulated 150 minutes of exercise per week had substantially lower risks of cancer mortality (i.e. HR=0.70 for all-cause death, HR=0.74 for all-cancer death). Another review study [22] synthesized and summarized evidence from 32 epidemiologic studies that evaluated exercise-cancer mortality associations in cancer patients, and showed significantly lower risks of cancer-specific mortality in cancer patients with higher exercise levels [22]. However, cautious interpretation is required in comparing the results from previously reported study due to the difference in definition between exercise and smoking. Physical activity is defined as any bodily movement produced by skeletal muscles that results in energy expenditure, while exercise is a subcategory of physical activity, which is defined as a planned, repetitive, and purposive in the sense that improvement or maintenance of one or more components of physical fitness is an objective [3].

In contrast to the previous study [20], the present study found J-curve associations, showing maximal levels of engagement in exercise were not associated with lowest levels of cancer risk. Additional research using more rigorous methodologies (e.g., repeated measures, objectively measured exercise information) is required to further explore the true associations between exercise and cancer in Asian populations.

The results of our study regarding breast, colorectal and prostate cancer were somewhat different with the results previously reported studies. In our study, breast cancer mortality between women who exercised, compared to those who did not exercise was not statistically significant 1.06 (0.75–1.50). Also, prostate cancer risk among men was not statistically significant (0.62, 0.32-1.50). However, both men and women showed decreased mortality among those who exercised for equal or more than 100 minutes per week. However, while previously reported studies were focusing on breast cancer, prostate cancer, colorectal cancer and other types of cancer, our study examined the association between exercise and cancer mortality on esophagus, lung, colorectal, and stomach cancer, thereby direct comparison between such studies are limited.

Results in our study generally demonstrated reducing cancer mortality for exercise for the above listed cancers. One thing was clear: 400-minute increase in exercise per week was not consistently associated with decreased mortality risk (Table 4).

A recent epidemiological study on exercise and all cause and cardiovascular disease mortality showed similar results as the present study in terms of identifying J-shaped associations [5, 23]. For example, the largest reduction in the risk of cancer mortality was observed in the middle categories of various running behaviors. As such, a seminal study also provided evidence on extremely high levels of exercise may not provide additional benefits for cardiovascular disease prevention [24]. However, no attention has been paid to disentangling the right amount of exercise for prevention of various types of cancer outcomes, in particular in Asian populations. In the present study using a large-scale prospective cohort of Korean adults, we provided convincing evidence for the first time that engaging in considerably high levels of exercise may not be associated with maximal reductions in risk of various cancer mortality outcomes. More research is clearly needed to confirm the J-shaped associations of various exercise indicators (including physical activity indicators) with each specific cancer outcome in Asian as well as Western populations.

To date, the mechanisms through which greater exercise levels lead to reduced risks of developing different types of cancer have been largely unknown. However, evidence suggests that engaging in exercise can cause favorable changes on body composition, sex hormone levels, systemic inflammation, and immune cell functions, all of which are important predictors of cancer [25]. Several previous studies have been proposed that may explain the mechanisms of reduction in cancer incidence or mortality by exercise [25, 26]. Natural killer cells are the most responsive immune cells to exercise, displaying an acute mobilization to circulation during physical exertion. In general, maximal mobilization of the Natural killer cells is achieved within 30 minutes of endurance training, after which continued exercise does not lead to further increase in Natural killer cell numbers [27].

There are several limitations. Information on exercise was obtained using a self-report method, so there may be measurement errors in the quantification of exercise levels due to recall bias and social desirability [28]. Self-reported activity data are deemed less accurate than objectively measured activity data using accelerometry [29]. However, contemporary accelerometers cannot distinguish exercise from physical activity although they can capture activity intensities. Hence, using self-reports in research (including the present analysis) to assess exercise as opposed to physical activity appears an inevitable, effective methodology. Another limitation is the use of data only from baseline. It is highly likely that individuals’ exercise levels and patterns change over time, but the present study was not able to take into account the variation in exercise in the analyses. Participants were recruited without employing any sampling strategy aimed at collecting a representative sample of adults. Hence, results from the present study may not be generalizable to the whole Korean adult population and adults from other countries. Also the participatns in our study had exercise and smoking data. Although physical activity involves various kinds of domain of physical activity and sedentary behavior which should be included in models to estimate mortality risk, our study only provided information on exercise. Among those who said that they had exercise, those who did not have information of the total duration of exercise per week was approximately 21%, and this might be regarded as an another limitation of our study. Our study also need to be interptreted carefully since those who exercise tend to be more interested in health, and as they are more likely to participate in health screening, they can lead to early detection of disease.

Especially, the longer the exercise period, the more pronounced this trend is. However, studies that have closely examined this mechanism are rare and need to be discussed in further studies.

## Conclusions

In conclusion, our study using a sample of over 300,000 Korean adults over 15 years of follow-up demonstrated that individuals who exercised showed considerably lower all-cause and cancer mortality risks than those who did no exercise. However, the associations of weekly exercise or duration with cancer mortality were J-shaped: all-cause and all-cancer death rates in both men and women with 400-minute increase in exercise per week. This observation indicates that risk of cancer mortality was lowest when participating in minimal or moderate levels of exercise, not in the highest level of exercise. Clinical trials and public health policies aimed at reducing cancer risk should encourage individuals to engage in exercise. However, cancer mortality risk may be minimized when such efforts are targeted for sedentary or physically inactive individuals to perform minimal or moderate levels of exercise.

## Additional file

Additional file 1: **Table S1.** List of cancers included in the study based on the International Classification of Diseases Tenth Revision codes. (DOCX 16 kb)

## Abbreviations

BMI: body mass index
CI: confidence interval
HR: hazard ratios
KMSMS: Korean Metabolic Syndrome Mortality Study
