# Supplementary material for: Exercise and cancer mortality in Korean men and women: a prospective cohort study
Source: BMC Public Health. 2018 Jun 19;18:761. doi: 10.1186/s12889-018-5669-1 (PMC6006742; doi:10.1186/s12889-018-5669-1)
Supplement: Supplementary file 1 — Table S1. List of cancers included in the study based on the International Classification of Diseases Tenth Revision codes. (DOCX 16 kb) [file 12889_2018_5669_MOESM1_ESM.docx]

Table S1: List of cancers included in the study based on the International Classification of Diseases Tenth Revision codes

| Site of cancer | ICD-10 | Description |
| --- | --- | --- |
| All cancer | C00-C97 |  |
| Esophagus | C15 |  |
| Head and neck | C00-06, C09-14, C32 | Oral cavity, Pharynx, Larynx |
| Liver | C22 |  |
| Lung | C34 |  |
| Colorectal | C18-20 |  |
| Pancreas | C25 |  |
| Kidney | C64 |  |
| Stomach | C16 |  |
| Prostate | C61 | Men only |
| Breast | C50 | Women only |
| Cervix | C53 | Women only |
